# Pediatrician's knowledge on the approach of functional constipation

**DOI:** 10.1016/j.rppede.2016.06.003

**Published:** 2016

**Authors:** Mario C. Vieira, Isadora Carolina Krueger Negrelle, Karla Ulaf Webber, Marjorie Gosdal, Sabine Krüger Truppel, Solena Ziemer Kusma

**Affiliations:** a Pontifícia Universidade Católica do Paraná, Curitiba, PR, Brazil; b Hospital Pequeno Príncipe, Curitiba, PR, Brazil

**Keywords:** Constipation, Pediatrics, Diagnostic, Treatment

## Abstract

**Objective:**

To evaluate the pediatrician's knowledge regarding the diagnostic and therapeutic approach of childhood functional constipation.

**Methods:**

A descriptive cross-sectional study was performed with the application of a self-administered questionnaire concerning a hypothetical clinical case of childhood functional constipation with fecal incontinence to physicians (n=297) randomly interviewed at the 36th Brazilian Congress of Pediatrics in 2013.

**Results:**

The majority of the participants were females, the mean age was 44.1 years, the mean time of professional practice was 18.8 years; 56.9% were Board Certified by the Brazilian Society of Pediatrics. Additional tests were ordered by 40.4%; including abdominal radiography (19.5%), barium enema (10.4%), laboratory tests (9.8%), abdominal ultrasound (6.7%), colonoscopy (2.4%), manometry and rectal biopsy (both 1.7%). The most common interventions included lactulose (26.6%), mineral oil (17.5%), polyethylene glycol (14.5%), fiber supplement (9.1%) and milk of magnesia (5.4%). Nutritional guidance (84.8%), fecal disimpaction (17.2%) and toilet training (19.5%) were also indicated.

**Conclusions:**

Our results show that pediatricians do not adhere to current recommendations for the management of childhood functional constipation, as unnecessary tests were ordered and the first-line treatment was not prescribed.

## Resumo

**Objetivo:**

Identificar o conhecimento do pediatra quanto ao manejo diagnóstico e terapêutico da criança com constipação intestinal funcional.

**Métodos:**

Estudo transversal descritivo com amostra constituída de médicos (n=297) entrevistados no 36º Congresso Brasileiro de Pediatria de 2013. Foi usado um questionário autoadministrado referente a um caso clínico hipotético de constipação intestinal.

**Resultados:**

Foi observada maior proporção de pediatras do sexo feminino, média de 44,1 anos, tempo de formação médio de 18,8 anos, 56,9% portadores de título de especialista pela Sociedade Brasileira de Pediatria. Exames complementares foram solicitados por 40,4%, a radiografia abdominal foi o mais requisitado (19,5%), seguido por enema opaco (10,4%), exames laboratoriais (9,8%), ultrassonografia de abdome (6,7%), colonoscopia (2,4%), manometria e biópsia (ambas 1,7%). Para o manejo foi sugerida a prescrição de lactulose (26,6%), óleo mineral (17,5%), polietilenoglicol (14,5%), suplemento de fibras (9,1%) e leite de magnésia (5,4%). Orientação alimentar (84,8%), desimpactação fecal (17,2%) e treinamento de toalete (19,5%) também foram indicadas.

**Conclusões:**

Evidencia-se uma discordância entre o manejo sugerido pelos pediatras e a conduta preconizada pela literatura disponível atualmente, uma vez que foram solicitados exames complementares desnecessários e não foi recomendada a orientação terapêutica considerada de primeira linha.

## Introduction

In clinical practice, intestinal constipation is a very common finding in children, corresponding to approximately 3% of consultations in general pediatric outpatient clinics and 25% of consultations in pediatric gastroenterology.^1^^-^ 4 When assessing the studies in Brazil, a variation of 14.7-38.8% was found in the prevalence of constipation.^5^ This wide variation is due both to the heterogeneity of the diagnostic criteria and the differences in study population selection.^5^^,^ 6

The Rome III Criteria (2006) states that in children older than 4 years, the diagnosis of constipation is established when there are ≤2 bowel movements per week; at least one episode of fecal incontinence per week; history of retentive posture or excessive voluntary stool retention; history of painful bowel movements; presence of a large fecal mass in the rectal canal; history of large-caliber stools that can clog the toilet bowl. The symptoms must be present at least once a week for a two-month interval.^4^ However, these criteria are considered by some experts as very restrictive.^5^ The European Society of Pediatric Gastroenterology, Hepatology and Nutrition (ESPGHAN) and North-American Society of Pediatric Gastroenterology, Hepatology and Nutrition (NASPGHAN) guidelines recommend using the Rome III criteria, except for symptom duration, since the recommended interval of two months can contribute to treatment delay in older children.^7^

The high prevalence of constipation generates high costs to public health, representing an expense of US$ 3362 per child treated annually in the United States.^6^ Studies have shown that there is no predominance of gender and in at least half of the cases constipation occurs in the first year of life, even if it is more often diagnosed in school age children.^1^^,^ 3

Complications associated with constipation include recurrent abdominal pain, fecal incontinence, rectal bleeding, enuresis and urinary infection/retention.^6^ These aggravating factors may progressively associate and negatively influence the quality of life, generating costs for both the family and the government.^2^^,^ 3

Although it is a disease with a relatively simple diagnosis and treatment, constipation affects the child's physical and emotional integrity.^1^ Taking into account the prevalence, clinical significance and impact of the disease, this study aims to outline a management panorama adopted by Brazilian pediatricians when treating a case of constipation and establish a parallel with the available literature.

## Method

This was a cross-sectional, descriptive study, with a sample of 297 physicians chosen by nonrandom convenience sampling, participating in 36th Brazilian Congress of Pediatrics, in Curitiba, state of Paraná, in October 2013. The congress participants were approached by individual researchers during the intervals of scientific activities and invited to participate and answer the questionnaire.

The study was approved by the Institutional Review Board of Pontifícia Universidade Católica do Paraná and written consent was obtained from all respondents. The study included pediatricians, general practitioners with a Specialist title in Pediatrics by the Brazilian Society of Pediatrics (SBP) and medical residents in Pediatrics. A self-administered questionnaire consisting of two parts was used as the research tool.

The first part, related to the respondent's identification, consisted of nine objective questions and aimed at drawing the respondent's medical profile, by gender, age, origin, time since graduation, extended education and place of work.

The second part of the questionnaire reported to the following fictitious clinical case: “J.L.C., male gender, six years old, has one bowel movement every three or four days, dry stools, pain and effort at evacuation. He soils his clothes three to four times a week. On physical examination: height 118cm and weight, 21.4kg. He shows palpable hardened feces in the left iliac fossa in moderate quantity. No more details”. This step contained discursive open questions concerning the diagnosis and initial clinical management, which were: I. What is the most probable diagnosis for this case? II. What are the criteria used for this diagnosis? III. Would you indicate any further examinations? If so, which ones? IV. What would be the therapeutic management of this patient? If you choose medication, which would be the drug and at what dose? A total of 412 questionnaires were distributed, of which 346 were answered. Of these, 49 questionnaires were excluded, as they were incomplete or illegible.

The Rome III criteria (2006) were considered for the diagnosis of functional constipation. The answers obtained from the questionnaires were transcribed and stored in a Microsoft Excel^®^ 2010 spreadsheet. Mean, median, minimum, maximum and standard deviation values were used to describe quantitative variables. Qualitative variables were described as frequencies and percentages. The chi-square test or Fisher's exact test was used to evaluate the association between the participants' profile variables and the prescription/indication variables. *P* values<0.05 were considered statistically significant. Data were analyzed using the IBM SPSS software v.20.0.

## Results

A total of 297 questionnaires were included. Among the interviewed population, there was a slight predominance of females (58.9%). The mean age was 44.1 years (23-75) and the mean number of the years since graduation was 18.8 (0-52). The majority of respondents came from the Southeast region (45.5%), followed by the South (26.3%), Central West (8.4%), Northeast (12.1%) and North (7.7%) regions. Most of the interviewed doctors (60.9%) had a Board Certification title by the Brazilian Society of Pediatrics (SBP), while the rest consisted of pediatricians without board certification and pediatric residents. Of the total, 89 doctors had a degree in the pediatric area, of which seven (2.4%) were pediatric gastroenterologists. General information about the study population is detailed in Table 1.

**Table 1:** Characteristics of respondents (n=297).

Variables	Description	n (%)
Gender	Female	175 (58.9)
	Male	122 (41.1)

Time since graduation	Mean	18.8 years

Region	North	23 (7.7)
	Northeast	36 (12.1)
	Midwest	25 (8.4)
	Southeast	135 (45.5)
	South	78 (26.3)

Educational background	Residency in Pediatrics with BCP^a^	170 (57.2)
	Residency in Pediatrics without BCP^a^	64 (21.6)
	Resident Physician (1st year)	27 (9.1)
	Resident Physician (2nd year)	25 (8.4)
	General Practitioner and BCP^a^	11 (3.7)

Pediatric specialty	Gastroenterology	7 (2.4)
	Others	82 (27.6)
	No	208 (70.0)

a BCP, Board Certification in Pediatrics granted by the Brazilian Society of Pediatrics/Brazilian Medical Association.

The diagnosis of constipation was identified by 93.6% of respondents. Complementary tests were indicated by 40.4%, with plain abdominal radiography being the most often requested test (19.5%). Barium enema (10.4%), abdominal ultrasonography (6.7%), anorectal manometry (1.7%), rectal biopsy (1.7%), colonoscopy (2.4%) and laboratory tests (9.8%) were also mentioned (Table 2).

**Table 2:** Diagnostic methods proposed by respondents.

Category	n (%)
*Need for complementary examinations*	
Yes	120 (40.4)
No	177 (59.6)

*Requested examinations*	
Abdominal radiography	58 (19.5)
Abdominal ultrasonography	20 (6.7)
Barium enema	31 (10.4)
Manometry	5 (1.7)
Biopsy	5 (1.7)
Colonoscopy	7 (2.4)
Laboratory tests	29 (9.8)

As for the management proposed by the respondents, it is noteworthy that 84.8% would make recommendations related to the patient's eating habits. Regarding other non-pharmacological measures, they also mentioned the use of additional fiber (9.1%) and toilet training (19.5%). A pharmacological approach was recommended by 64% of respondents and included the use of lactulose (26.6%), mineral oil (17.5%), polyethylene glycol (PEG) (14.5%) and magnesium hydroxide (5.4%). None of the responses recommended the use of more than one laxative simultaneously. Fecal disimpaction was suggested by 17.2% of the interviewed physicians (Table 3).

**Table 3:** Therapeutic management proposed by respondents.

Suggested treatment	n (%)
Fecal disimpaction	51 (17.2)
Polyethylene glycol	43 (14.5)
Lactulose	79 (26.6)
Mineral oil	52 (17.5)
Magnesium hydroxide	16 (5.4)
Fiber supplement	27 (9.1)
Nutritional guidance	252 (84.8)
Toilet training	58 (19.5)

**Figure 1:**
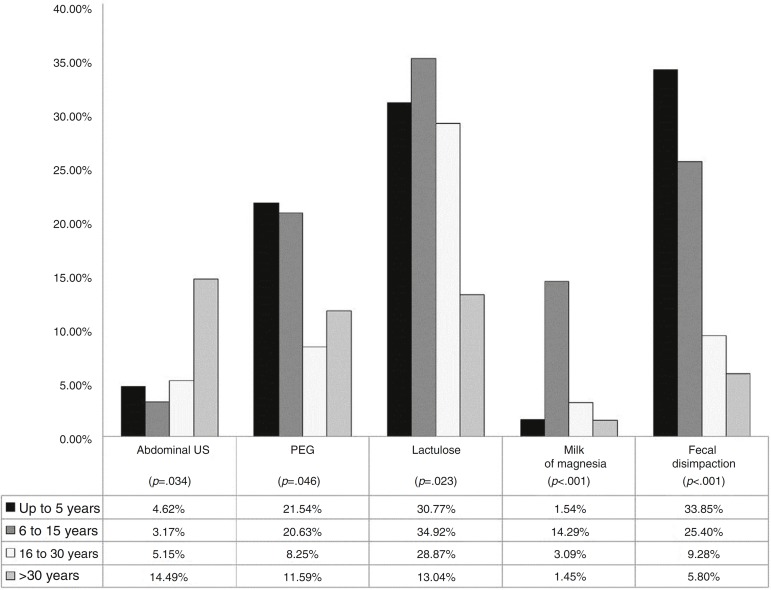
Association between time since graduation and diagnostic and therapeutic procedures.

An association was identified between the time since graduation and certain conducts in the diagnostic and therapeutic management (Fig. 1). Among the doctors that had graduated less than six years before, 4.6% would request an abdominal ultrasound. In contrast, 14.4% of those that had graduated more than 30 years before would do the same (*p*=0.034).

Regarding the use of polyethylene glycol, it was indicated by 21.5% of doctors that had graduated less than six years before and 20.6% of those graduated between 6 and 15 years before, with a lower percentage of prescription among the ones that had graduated more than 15 years before (*p*=0.046).

Approximately 30% of each group of respondents with time since graduation less than 30 years prescribed lactulose. Respondents that had graduated between 6 and 15 years prescribed magnesium hydroxide at a higher percentage (14.3%) when compared to the other groups of time since graduation. This drug was prescribed by 1.4% of professionals that had graduated more than 30 years before, by 1.5% among those with less than 5 years since graduation and 3.1% among those that had graduated 16-30 years before (*p*<0.001). Among those that had graduated less than 6 years before, 33.9% indicated fecal disimpaction, as 25.4% of those graduated 6-15 years before; 9.3% of those graduated 16-30 years before and 5.8% of those that had graduated more than 30 years before (*p*<0.001).

## Discussion

There can be several etiologies for constipation. Functional constipation is the most common, in which the most important originating factor seems to be voluntary fecal retention.^2^^,^ 4 Organic causes of constipation include metabolic disorders (hypothyroidism, cystic fibrosis, hypercalcemia and hypocalcemia), neuropathic (Hirschsprung's disease, myelomeningocele, spina bifida, cerebral palsy) and immunological diseases (allergy to cow's milk protein, celiac disease), as well as the use of medications such as iron salts, antacids, anti-inflammatory drugs and opioids.^3^^,^ 5

The Rome III Criteria (2006) is the one most often used to determine constipation in childhood.^1^^,^ 7 ^-^ 9 In the aforementioned clinical case, symptom duration was not stated (if>2 months), a factor that can be considered a study limitation. The ESPGHAN and NASPGHAN guidelines recommend that the Rome III criteria be used to define the presence of constipation, except for symptom duration, considering that the two-month interval recommended for older children can contribute to treatment delay.^7^

The practical guide proposed by National Institute for Health and Clinical Excellence (NICE) states that a detailed history and a thorough physical abdominal examination are enough to attain the diagnosis.^10^ This is also the final conclusion of the guidelines proposed by ESPGHAN and NASPGHAN, suggesting that the diagnosis is primarily clinical and that complementary tests are not necessary to confirm the disease.^7^ Nevertheless, 40.4% of the respondents deemed necessary to request additional tests.

A plain abdominal radiography may be useful to characterize fecal impaction in children and to evaluate the effectiveness of the initial treatment.^11^ However, despite being requested by 58 physicians (19.5%), it is not considered necessary to attain a final diagnosis, as there is no association between the presence of suggestive symptoms and the accumulation of stool in the rectum.^8^ It is noteworthy that there are no studies associating the need for abdominal radiography when the patient does not meet the clinical criteria for constipation.^7^^,^ 11 ^,^ 12

Abdominal ultrasound was another very often-indicated test. Twenty professionals (6.7%) found it necessary to request this examination to confirm the diagnosis. In spite of being seen as a simple, noninvasive method for evaluation of fecal retention and measurement of the transverse rectal diameter, it is not considered an essential examination.^1^^,^ 7 ^,^ 13 To date, there is not enough evidence establishing that an alteration in rectal diameter can be used as a predictor for the presence of constipation in children.^11^^,^ 14

The barium enema was indicated by 10.4% of respondents. According to the literature, this is one of the main tests used to rule out the most important differential diagnosis for functional constipation in children, Hirschsprung's disease.^15^^,^ 16 However, this test is not required for the final diagnosis, unless the child has suggestive signs, such as an early onset of constipation, delayed passage of meconium and significant abdominal distension.^7^^,^ 15

The same is true for anorectal manometry (ARM), indicated by five of the interviewed physicians (1.7%). This test also aims to rule out Hirschsprung's disease by disclosing alterations in the anorectal inhibitory reflex.^17^^-^ 20 However, it is noteworthy that the examination is not essential for the diagnosis, especially in children after the neonatal period.^7^ As with the barium enema, ARM should only be performed if there are data suggestive of Hirschsprung's disease and in severe cases refractory to adequate treatment.^7^^,^ 17 ^,^ 20 The rectal biopsy is considered the gold standard for the diagnosis of Hirschsprung's disease and should only be performed when the tests discussed above suggest its presence.^12^ It should not be performed at the patient's initial consultation, unlike what was proposed by seven professionals interviewed in this study (2.4%).^7^

Colonoscopy was another examination suggested by the interviewed physicians (1.7%). As with the analysis of colonic transit, the colonoscopy should not be indicated at the initial consultations.^7^

Finally, laboratory tests were requested by 29 pediatricians (9.8%). Such tests may be indicated when there is diagnostic uncertainty (patient does not meet the clinical criteria), or when there is strong suspicion of an underlying organic disease, such as hypothyroidism, allergy to cow's milk protein and celiac disease and not for patients with a clear picture of functional constipation, as in this study case.^1^^,^ 7 ^,^ 10 ^,^ 15

The hypothesis of functional constipation^2^ should prevail in the management of the child with constipation, if there are no data in clinical history and physical examination suggesting a secondary cause. Traditionally, the therapeutic management consists of four steps, including general recommendations and education, disimpaction when necessary, re-impaction prevention and retraining of bowel habits.^4^ When transmitting the general recommendations, it is important to establish a cooperative relationship between the physician and the family, including the patients, when their age allows it.^2^^,^ 7 ^,^ 21 The basic understanding of the pathophysiology involved helps to ease family tensions and feelings of insecurity or guilt.^2^^,^ 21

A balanced diet, including the consumption of whole grains, fruits and vegetables, together with an adequate water intake, is recommended as part of the maintenance treatment for constipation in children.^4^ Among the respondents, 84.8% suggested some type of nutritional guidance. There are conflicting reports in the literature about the role of fiber intake in children with constipation. However, supplementation with soluble fiber, considered by 9.1% of the pediatricians, has not shown sufficient efficacy and its use is not indicated.^4^^,^ 7

The toilet training goal should be to retrain the bowel habits.^2^^,^ 4 The child should be advised to remain sitting on the toilet bowl for 5-10min after the main meals, in an adequate position for the abdominal press, while using a footrest.^1^^,^ 4 Among the interviewed physicians, 19.5% made recommendations about patient toilet training.

The elimination of the fecaloma through fecal disimpaction should be performed when the presence of a mass is identified during abdominal palpation, rectal examination or plain abdominal radiography.^4^ The disimpaction can be made with enemas or laxatives, such as polyethylene glycol (PEG), with both being of similar efficacy.^22^ As the proposed clinical case showed evidence of fecal retention on the physical examination, fecal disimpaction would be indicated. However, the procedure was considered by only 17.2% of the pediatricians. None of the respondents specified the type of fecal disimpaction (orally or by enema). It should be noted that the disimpaction before the maintenance therapy is recommended to increase treatment success and reduce the risk of fecal incontinence.^1^^,^ 4 ^,^ 18 Once the disimpaction is performed, the focus of the treatment should be recurrence prevention with the use of maintenance medications.^1^^,^ 21

The pharmacological approach was recommended by 64% of respondents. Among the drugs used to treat constipation, polyethylene glycol (PEG) is the most effective when compared to lactulose, magnesium hydroxide, mineral oil or placebo.^7^ PEG is a compound of high molecular weight, poorly absorbed by the body and not metabolized by intestinal bacteria.^17^^,^ 23 It exerts an osmotic, non-irritating action, with consequent increase in the water content of stools.^17^^,^ 23 It should be considered as the first-line maintenance treatment of intestinal constipation.^7^^,^ 23 However, it was prescribed by only 14.5% of the respondents. A considerably higher number of pediatricians (26.6%) indicated the use of lactulose. Several studies comparing the use of PEG and lactulose suggested the superiority of PEG treatment, higher success rates in the treatment and prevention of fecal impaction recurrence, greater relief of abdominal pain and fewer side effects.^24^^-^ 27 The maintenance treatment with lactulose is recommended when PEG is not available.^7^ Therefore, an inversion in the prescription rates of PEG and lactulose can be observed in the available literature. The prescription of magnesium hydroxide, considered by 5.4% of pediatricians and of mineral oil, prescribed by 17.5%, should be used as adjunctive therapy or second-line treatment of constipation.^7^

Early adequate treatment of constipation is essential to prevent complications, such as fecal incontinence, described in our case. When analyzing the conducts taken by the respondents, one should consider the location where each professional works, taking into account possible differences in the availability of resources, both diagnostic and therapeutic. Some statistically significant associations (*p*<0.05) were observed between the respondent's profile and some of the approaches used. The professionals that had graduated more than 30 years before were more likely to request an abdominal ultrasound; 14.5% of this group requested this test, considered unnecessary for the disease diagnosis. In contrast, this same group was the one that showed the least number of lactulose prescriptions in patient management: only 13.4% chose this drug, *versus* 34.9% of professionals that had graduated 6-15 years before (Fig. 1). Polyethylene glycol and fecal disimpaction, considered the first choice for the patient presented in the study, were more often indicated by physicians that had less time since graduation.^1^^,^ 2 In relation to PEG, it was prescribed by 21.5% of physicians that had graduated less than six years before and 20.6% of physicians that had graduated 6-15 years before. As for fecal disimpaction, it was indicated by 33.8% of the ones that had graduated less than six years before and 25.4% of those graduated 6-15 years before (Fig. 1). Doctors that had graduated between 6 and 15 years before were the ones that most often prescribed magnesium hydroxide (14.3%), which is considered a second-line or adjunctive therapy drug (Fig. 1). These results demonstrate the great disparity in the type of management used by physicians with longer time since graduation and those with less time, which reinforces the need for constant updating by health professionals.

Recent studies, published after data collection was finished for this study, showed differences in behaviors proposed by general practitioners, pediatricians and pediatric gastroenterologists.^28^^,^ 29 A study carried out in Saudi Arabia, which used a questionnaire with questions about practical and demographic characteristics, definition, management and treatment of constipation, showed that pediatricians more often prescribed the use of lactulose and indicated fecal disimpaction when compared to general practitioners.^28^ A national study performed in 2009 with physicians in the state of Minas Gerais, showed that 72.6% of pediatric gastroenterologists requested additional tests, when compared with 27.5% of the other interviewed physicians.^29^ The study also disclosed that the most often recommended drugs by non-gastroenterologists were mineral oil (72.6%), magnesium hydroxide (52.1%), lactulose (41%) and PEG (25.2%). Among the pediatric gastroenterologists, the most often recommended drugs were magnesium hydroxide (91.7%), PEG (91.7%) and mineral oil (58.3%). These studies included the administration of a questionnaire on constipation, in comparison with the present study, which used an open clinical case as part of the research tool.

The limitations of the present study include the fact that it is a cross-sectional one, that it identified the physician's circumstantial idea in relation to a specific case, and the inclusion of physicians who were pediatric residents. One should also consider that the selection of respondents by convenience has the advantage of evaluating an accessible population, but results in the incapacity to make statements that can be strictly generalizable. The results obtained might depict a good image of the conduct suggested by the pediatricians; however, it is not possible to use statistical tools to measure the accuracy of results.

Despite the abovementioned limitations, the inadequacy in diagnostic and therapeutic procedures demonstrated in this study highlights the need for continuing education programs in order to update pediatricians regarding the management of intestinal constipation. The mistakes made in diagnostic management subject the patient to unnecessary tests, which are often invasive and do not influence the recommended approach. The use of therapies considered as second-line ones as the first treatment option may result in treatment failure or refractory disease. Treatment delays and inadequate treatments may result in the onset of complications, negatively influencing the child's quality of life and generate costs for both the family and the health system.
